# *In vivo *dose-response of insects to Hz-2V infection

**DOI:** 10.1186/1743-422X-1-15

**Published:** 2004-12-21

**Authors:** John P Burand, Christopher P Rallis

**Affiliations:** 1 Department of Entomology, University of Massachusetts at Amherst, Amherst, Massachusetts, USA; 2 Department of Microbiology, University of Massachusetts at Amherst, Amherst, Massachusetts, USA

## Abstract

**Background:**

Hz-2V infection of female *Helicoverpa zea *moths is manifested as insects that are either sterile "agonadal" individuals with malformed reproductive tissues or fertile asymptomatic carriers which are capable of transmitting virus on to their progeny. Virus infected progeny arising from eggs laid by asymptomatic carrier females may themselves be either sterile agonadals or asymptomatic carriers.

**Results:**

By injecting virus into female moths, a correlation was established between virus doses administered to the females and the levels of resulting asymptomatic and sterile progeny.

**Conclusions:**

The results of these experiments indicate that high virus doses produced a higher level of agonadal progeny and lower doses produced higher levels of asymptomatic carriers.

## Background

The insect virus, Hz-2V originally named gonad-specific virus (GSV) [1] was first identified in moths from a colony of corn earworms, *Helicoverpa zea *originating at the USDA-ARS in Stoneville, MS [1,2]. Insects infected with this virus were found to have malformed and missing reproductive tissues and were sterile, a condition that has been referred to as "agonadal". The examination of infected moths revealed that this virus replicated in a variety of male and female reproductive tissues including the common and lateral oviducts. Hence the tropism and replication of the virus is not specific to gonadal tissues. This rod shaped, enveloped, DNA virus has been more appropriately named Hz-2V since it resembles Hz-1V in size, pathology *in vitro *and in genome structure and size [3-5].

While examining progeny from eggs laid by infected female moths, Hamm *et al*. [2] identified individuals that appeared healthy and were capable of transmitting Hz-2V to their progeny. Using PCR analysis Lupiani *et al*. [6], were able to detect viral DNA sequences in feral corn earworms from wild populations that appeared healthy. These apparently healthy, infected moths are asymptomatic carriers of Hz-2V. The ability of this virus to persist in these asymptomatic carriers is a key feature of the biology of this virus. Since productive replication of Hz-2V results in the gross malformation of reproductive tissues and sterility of infected adult moths, persistence in asymptomatic carrier moths allows the virus to be maintained in insect populations such as the Stoneville colony.

Hamm *et al*. [2] presented evidence from experimental matings involving asymptomatic female moths and uninfected males that showed the proportion of agonadal progeny arising from eggs laid on successive oviposition days increased rapidly with each oviposition day, suggesting a change in viral activity in the asymptomatic female. They proposed that the outcome of virus infection in progeny was related to virus dose, such that eggs laid on early oviposition days received a low virus dose resulting in more asymptomatic virus carrier moths, whereas those arising from later oviposition days received a high virus dose and developed into agonadal moths. These findings indicate that Hz-2V is able to exist in a persistent or latent state in some corn earworms and become induced into productive replication at a specific time in the development of the insect. During their experiments, Hamm *et al*. [2] were unable to accurately determine and control the virus dose female moths received and they were unable to directly detect females that were asymptomatic carriers of the virus.

Raina *et al*. [7] showed that it was possible to inject Hz-2V into healthy female corn earworm moths, and upon mating with healthy male moths, produce asymptomatic carrier and agonadal progeny. They found that about half of all of the progeny produced by females that were infected with a moderate virus dose exhibited the agonadal condition and that about 90% of the remaining apparently healthy progeny actually carried viral DNA sequences detectable by PCR. This data suggests that adult females can be injected with virus to experimentally produce females that mimic the asymptomatic carrier females described by Hamm *et al*. [2].

In this study we have used the approach of injecting virus into healthy female moths to examine the relationship between virus dose and the level of infected, agonadal and asymptomatic carrier progeny insects hatching from eggs laid on successive ovipostion days. The results presented here demonstrate that virus dose affects both the level of infected progeny and the kind of infection found in insects hatching from eggs laid by virus infected females, indicating a direct correlation between virus dose received by females and the level of infected progeny they produce. Also demonstrated here is the fact that for each virus dose, as the level of agonadal insects hatching from eggs laid on successive oviposition days increase, the level of asymptomatic carrier progeny decreases.

## Results

**Figure 1:**
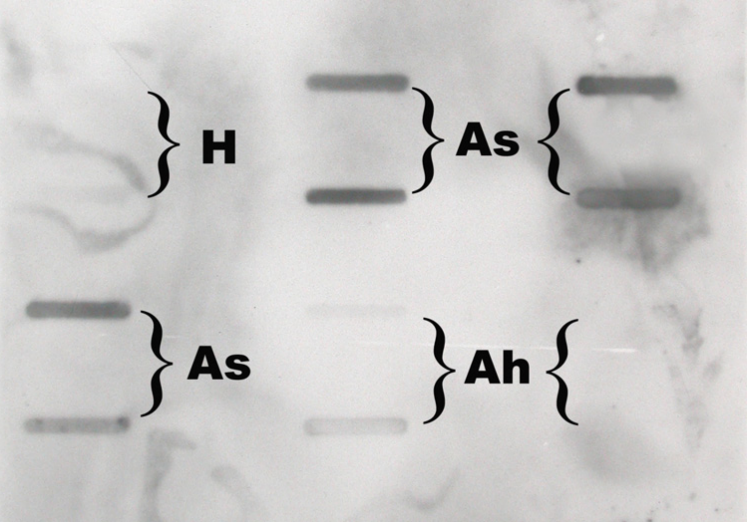
Slot blot hybridization results of DNA extracted from reproductive tissues of corn earworm moths. DNA was extracted, amplified via PCR, transferred onto a nylon membrane, and hybridized with a DIG-labeled viral DNA probe. Dark blots are indicative of DNA from asymptomatic carrier moths (As). Blots of DNA samples from insects from the healthy colony (H) and from insects that were determined to be apparently healthy (Ah) were blank or very light.

**Figure 2:**
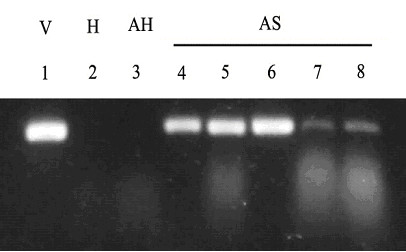
Agarose gel of PCR products from DNA extracted from the reproductive tissues of corn earworm moths. The first lane is from a sample containing purified Hz-2V DNA (V). Lanes 2 denotes a sample from normal, healthy moth (H) from our insect colony. Lane 3 contains a DNA sample from an apparently healthy (AH) progeny moth arising from and infected female. Lanes 4–8 contain DNA samples extracted from asymptomatic progeny corn earworm moths (AS).

A total of 1856 progeny moths resulting from approximately116 eggs laid on each of the first four oviposition days by females infected with 2 × 10^5^, 2 × 10^6^, 2 × 10^7^, or 2 × 10^8 ^TCID_50 _units of Hz-2V were dissected and the reproductive tissues examined for signs of virus pathology. The PCR products of DNA samples from reproductive tissues of all apparently healthy progeny moths were examined for the presence of Hz-2V DNA via slot blot hybridization (figure 1), and the size of the PCR products of representative samples was determined by agarose gel electrophoresis. The results of agarose gel electrophoresis of PCR products from representative samples of agonadal, asymptomatic carriers and apparently healthy moths are shown in figure 2.

Moths that had reproductive tissues that appeared to be normal but tested positive for Hz-2V DNA by PCR analyses were considered asymptomatic carriers of the virus. For each virus dose tested the number of agonadal moths, asymptomatic carriers, infected individuals (the sum of agonadal and asymptomatic carriers), and uninfected progeny moths hatching from eggs laid on each oviposition day was recorded.

**Figure 3:**
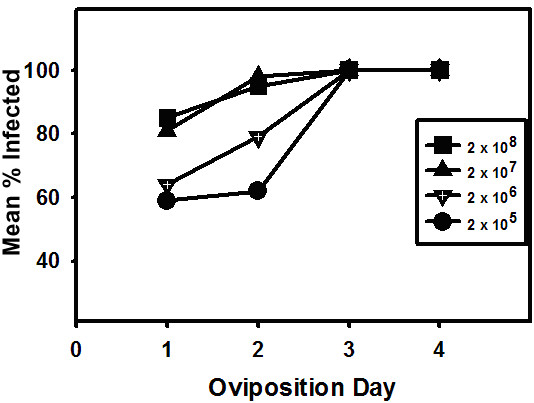
Mean percentages of infected (agonadal and asymptomatic carriers) progeny arising from eggs laid by female moths infected with 2 × 10^5^, 2 × 10^6^, 2 × 10^7^, or 2 × 10^8 ^TCID_50 _units of Hz-2V.

The analysis of these results showed that the percentage of total infected progeny (asymptomatic carriers and agonadal moths) at all doses tested increased with each successive oviposition day, and the level of infected progeny increased as virus dose increased from 2 × 10^5 ^to 2 × 10^8 ^TCID_50 _units (figure 3). For individuals hatching from eggs on oviposition day one, the highest percentage of infected progeny (approximately 80%) was produced by females infected with the two highest virus dose (2 × 10^7 ^and 2 × 10^8^), whereas the lowest percentage (about 60%) was produced by females infected with the lowest doses of virus (2 × 10^5 ^and 2 × 10^6 ^TCID_50_).

**Figure 4:**
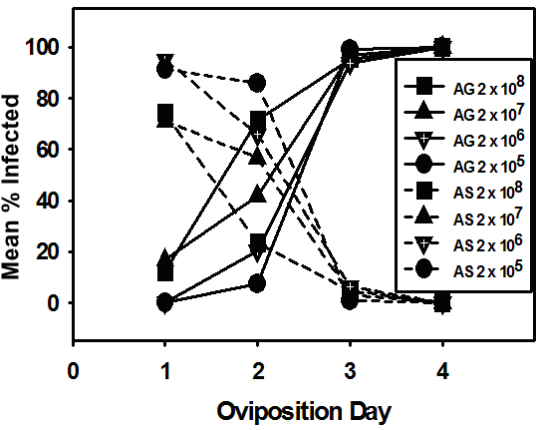
Mean percentages of all (male and female) agonadal (AG) and asymptomatic carrier (AS) F1 moths arising from eggs laid by females infected with 2 × 10^5^, 2 × 10^6^, 2 × 10^7^, or 2 × 10^8 ^TCID_50 _units of Hz-2V.

Virus infected progeny moths arising from eggs laid on each oviposition day by females infected with different virus doses were divided into agonadal and asymptomatic carriers and these results are presented in figure 4. No agonadal insects arose from eggs laid on oviposition day one by females infected at the lowest virus doses, whereas approximately 15% of the progeny females from eggs laid at this time by females infected at the two highest doses were agonadal. At all of the viruses doses tested, between 70 and 90% of the individuals hatching from oviposition day one eggs were asymptomatic carriers (figure 4).

For all F1 insects hatching from eggs laid on day two, (figure 4) the percentage of agonadal moths increased with increasing virus dose and the percentage of asymptomatic carriers at each dose declined (figure 4). The highest number of agonadal moths (approximately 70%) hatching from eggs laid on day two came from females that received the highest virus dose. At the two lowest doses the level of agonadals hatching from day two eggs was between 5 and 20%. At all doses almost 100% of the eggs laid on days three and four gave rise to agonadal moths.

**Figure 5:**
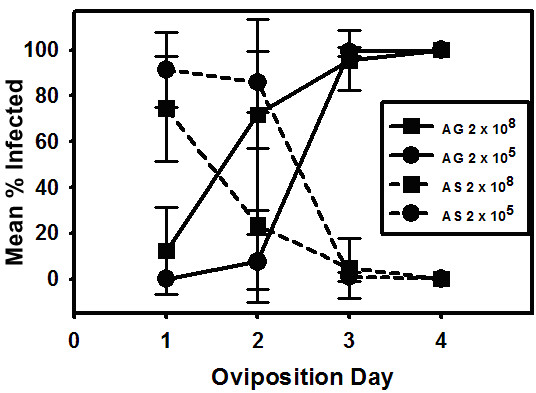
Mean percentages of all (male and female) agonadal (AG) and asymptomatic carrier (AS) F1 moths arising from eggs laid by females infected at the highest and lowest doses of Hz-2V.

In order to better illustrate the relationship between the two types of infections and to emphasize the effects of virus dose upon the proportions of asymptomatic and agonadal infections, percentages of asymptomatic carriers and agonadal progeny for only the highest and lowest dose are presented in figure 5. The trend in the two types of infected progeny insects follows the same general pattern for both virus doses relative to oviposition day. That is, at both doses the percentage of agonadal progeny increases with ovipostion day as the percentage of infected insects that are asymptomatic carriers of Hz-2V decreases. At the highest dose, the proportion of agonadal insects starts out higher (~ 10%) on the first oviposition day than that of agonadal progeny of females infected at the lowest dose (0%), and rises more quickly to ~ 70% of the progeny from eggs laid on oviposition day two. This is compared to only about 5% of the progeny arising from day two eggs laid by females infected at the lowest dose. Interestingly the reverse is the case for asymptomatic progeny hatching from oviposition day one eggs. Whereas approximately 90% of the infected oviposition day one individuals from females infected at the lowest virus does are asymptomatic only about 10% of the individuals from females infected at the highest dose are asymptomatic.

## Discussion

Injecting Hz-2V into female moths results in experimentally infected insects that resemble asymptomatic females and females that have become infected with the virus during copulation, not unlike the females infected during mass-matings by infected males in transmission experiments conducted by Hamm *et al*. [2]. These infected moths appear healthy, are fertile, and can transmit the virus to progeny that result from mating. Some of the progeny moths arising from these infected females do not carry any detectable Hz-2V DNA sequences, others are sterile with malformed reproductive tissues, and still others are fertile, asymptomatic carriers of the virus. This variety of infections suggests that the virus is not initially present in all of the eggs laid by infected females, but is transmitted transovarially to some of the eggs at sometime time prior to oviposition. This idea is important in that it suggests that the dose or titer of virus transmitted from the parent female moth to the developing oocytes is not constant, and that the virus dose that each oocyte receives determines the outcome of the infection when these progeny insects mature into adult moths. The precise molecular mechanism that determines which infected individuals become agonadal and which will maintain the virus in the population as asymptomatic carriers has yet to be determined.

The results presented in figure 4, demonstrate that the percentage of agonadal progeny resulting from eggs laid on oviposition day one by female moths infected with Hz-2V increased as the dose of Hz-2V used to infect female moths increased. Progeny arising from eggs laid on oviposition day two also exhibited this correlation between virus dose and percent agonadal progeny. This indicates that the titer of the virus present in the experimentally infected female moths determines the amount of virus that is transmitted to eggs, and is directly correlated to the percentage of agonadal progeny arising from eggs laid by the infected females. As the dose of Hz-2V used to infect a female moth is increased, a corresponding increase is observed in total agonadal progeny arising from all eggs laid by the infected female.

The percentage of agonadal progeny also increases with each successive oviposition day, approaching 100% agonadal progeny by day three at all virus doses tested, and all progeny moths arising from eggs laid on oviposition day four in all groups were agonadal. Based on the correlation between virus titer and percent agonadal progeny observed in these experiments, the increase in agonadal progeny per oviposition day is likely due to an increase in the titer of virus transmitted to the eggs, suggesting that the titer of virus increases in the parent female moths with each successive oviposition day.

Studies of Hz-2V replication *in vitro *revealed a rapid increase in virus titer by 24 hours post infection in Tn-368 and Ld-652Y cells [4,5]. Hz-2V replication *in vivo *in the epithelial cells of agonadal female oviduct tissue has been described previously by Rallis and Burand [8]. The level of detectable virus in these tissues increased dramatically between 8 days post pupation (dpp), measured from the day the last larval exuviae was shed, and 10 dpp. It is likely that the large increase in virus over a 24 hour cycle observed *in vitro *also occurs *in vivo*, resulting in a significant daily increase in the titer of Hz-2V in these experimentally infected female moths. Although the precise site of virus replication in these experimentally infected females is not known, the increase in virus titer in these individuals almost certainly results in an increase in virus being transmitted to the progeny with each successive oviposition day and ultimately in the patterns of infection reported here.

If, as we have proposed, low virus doses result in asymptomatic carrier moths, and high virus doses produce agonadal progeny, then asymptomatic carrier progeny would likely arise from eggs produced on the earliest oviposition days and decrease with each day, as the virus titer in the egg-laying female moth increases. In fact, the percentage of asymptomatic carrier progeny in these experiments does decrease with each successive oviposition day to 0% by day four. The percent asymptomatic carriers is highest in progeny that receive the lowest virus dose, specifically progeny from oviposition day one and progeny arising from the parent female moths that were experimentally infected with the lowest dose of virus. This is directly opposite of what is observed for agonadal progeny, which is at its highest level at the highest virus dose, specifically on the later oviposition days (days three or greater) and in progeny arising from parent female moths that were infected with the highest virus dose. Interestingly, the lowest percentage of asymptomatic carrier progeny arose from eggs laid by the group of female moths that received the highest virus dose of Hz-2V (figure. 3). These data suggest that the virus dose transmitted by infected female moths to their developing eggs determines whether the progeny develop the agonadal condition or become asymptomatic carriers of Hz-2V.

The results presented here clearly show that there is a direct correlation between virus dose and the relative percentage of agonadal and asymptomatic progeny. That is, increasing the virus dose causes an increase in the percentage of agonadal progeny, but a decrease in the percentage of asymptomatic progeny. At the present time, it is unknown how the development of an infected individual into an agonadal adult or an asymptomatic carrier is regulated. It is likely that a minimum titer of Hz-2V is needed at a key point in larval development to produce a viral factor(s) within the larval tissues at a threshold level required to reprogram the development and differentiation of the reproductive tissues into the agonadal structures. If this threshold is equaled or exceeded at this point in development, the progeny will exhibit the agonadal condition. However, if this threshold level is not attained, then the reproductive tissues are not reprogrammed and the infected insect becomes an apparently healthy, fertile, asymptomatic carrier of Hz-2V.

## Conclusions

The evolution of Hz-2V infection in *H. zea *has resulted in the ability of the virus to produce two different types of infections in the insects that enable the virus to replicate to high titers in the reprogrammed reproductive tissues in sterile agonadal moths, while maintaining itself in a population in asymptomatic carrier moths. This replication strategy appears to be essential for the continued existence of Hz-2V, since the development of the sterile, agonadal condition in *all *infected moths would lead to the extinction of the insect host, and the possibly the virus as well. The production of asymptomatic carrier moths ensures that some fertile, infected moths exist that can mate and produce infected progeny, enabling an Hz-2V-infected population to sustain itself, as in the case of the Stoneville colony.

## Methods

### Source of insects and virus

Corn earworm larvae used to start a laboratory colony of healthy *H. zea *were obtained from the USDA-ARS in Stoneville, MS. Insects were reared on artificial diet and maintained as outlined previously [9].

Hz-2V for infecting female moths was prepared as described previously and purified via sucrose gradient centrifugation [4].

### Injection of adults

Newly emerged adult female moths were prepared and injected with Hz-2V as outlined by Rallis and Burand [8]. The female moths were divided into four dose groups, and 9 or 10 insects were infected with Hz-2V at one of the following concentrations of 2 × 10^5^, 2 × 10^6^, 2 × 10^7^, and 2 × 10^8 ^TCID_50 _units.

### TCID50 assays

Tn368 cells were cultured as per Burand & Lu [4] and 100 ul of cell culture medium containing 8 × 10^4 ^Tn368 cells were seeded into each well of a 96-well plate. Between 6 and 13 serial dilutions were made from each virus sample assayed and 10 or 20 wells were plated with 10 ul for each dilution. Plates were incubated at 27°C for 3 to 4 days and examined for the appearance of cytopathic effect (CPE). The numbers of wells with CPE were counted and the TCID_50 _calculated [9].

### DNA extraction and purification of viral DNA

DNA was extracted from the reproductive tissues of adult moths by first homogenizing dissected tissues in 200 ul of TE buffer (10 mM Tris, pH 7.4, 1 mM EDTA, pH 8.0) followed by a 2-minute incubation in a boiling water bath. The homogenate was then chilled on ice, after which Ribonuclease A (10 ug/ul) was added to each sample, which was then incubated at room temperature for 15 min. The samples were then clarified by centrifugation at 15,600 × g for 2 min.

Viral DNA used as template for PCR reactions was extracted from purified virus using 1% SDS in TE containing 1 mg/ml Protease K as outlined by Burand and Lu [4].

### PCR amplification of viral DNA sequences

Two sets of primers were used to amplify Hz-2V genomic DNA to prepare a probe for use in slot blot analysis of insect reproductive tissues. The first set (P4-1, 5'-GCACGATTCGTAATGTTC-3'; and P4-2, 5'-GCACACCTATCAATCACC-3') was designed to amplify a 434 bp sequence of the Hz-2V genome [6]. PCR reactions using P4-1 and P4-2 primers were brought to a final volume of 20 ul using the Bioneer AccuPower^® ^PCR reagent premix kit with 1 unit of Taq DNA polymerase. Each reaction was carried out in10 mM Tris-HCl (pH 9.0), 1.5 mM MgCl_2 _and 40 mM KCl, containing 250 uM of each of the four dNTP's, with 100 pM of P4-1 forward and P4-2 reverse primers, and 10 ng of purified viral DNA as template. These primer set and reaction conditions were also used to amplify viral DNA sequences in approximately 100 ng of DNA from reproductive tissues of moths thought to be asymptomatic carriers of Hz-2V.

The second set of primers (P4-3, 5'-GCTGTGCTGTACAAGTGC-3'; and P4-4, 5'-CCCTTGACGATCCCTTTTG-3') was designed to amplify a 350 bp region directly interior to that of the P4-1 and P4-2 amplified sequence. These primers were used to generate a DIG-labeled probe for Hz-2V to be used in slot blot hybridization assays. PCR reactions for production of the DIG-labeled probe were carried out in a final volume of 50 ul using the Boehringer Manheim DIG High Prime DNA Labeling and Detection Kit, with 1X concentrations of Taq Polymerase buffer (100 mM Tris-HCl pH 8.0, 500 mM KCL pH 8.3, and 25 mM MgCL_2_), 100 pM of both P4-3 and P4-4 primers, a hexanucleotide mixture containing DIG-labeled dUTP (2 mM dATP, dCTP, dGTP, 1.3 mM dTTP, and 0.7 mM alkali labile DIG-11 dUTP pH 7.0), and 100 pM of Hz-2V genomic DNA. The DIG-labeled PCR product was purified on a 0.8% agarose gel using the Qiagen gel electrophoresis purification kit.

Both PCR reactions for amplification of the viral DNA in tissue samples and for the production of the viral DNA probe consisted of 30 cycles of a DNA denaturation step at 95°C for 1 min., a primer annealing step for 1 min. at 55°C, and a 1 min. primer extension step at 72°C.

### Detection of a viral DNA sequence by slot blotting

To prepare the DNA for slot blot analysis, 15 ul of the P4-1 and P4-2 PCR amplified DNA from insect samples was denatured by incubating with NaOH (0.4 M)/ EDTA (10 mM, pH 8.2) at 100°C for 10 min., then applied to a Hybon-N+ membrane prewashed with 500 ul 5X SSC buffer (0.6 M NaCl, 60 mM Na citrate pH 7.0) in a Manifold II slot blotter (Schleicher & Schuell). After applying the DNA, the membrane was baked at 88°C for 2 hrs under vacuum and prehybridized for 6 hrs. at 42°C in 50% formamide prehybridization buffer (5X SSC, 0.1% (w/v) N-laurylsarcosine, 1% (w/v) Na_2_-Dodecylsulfate, 2% Blocking reagent (Boehringer-Manheim), and 50% Formamide). Slot blots were hybridized with 150 ng DIG-labeled Hz-2V probe at 37°C for 12–14 hrs. Following washing, chemiluminescent detection was carried out as recommended by the DIG High Prime Labeling and Detection Kit Manual for DNA Hybridization (Boehringer Mannheim).

### Analysis of PCR products by agarose gel electrophoresis

In order to confirm that the PCR products that hybridized to the viral DNA probe contained an amplified DNA fragment of the appropriate size (434 bp), representative samples were analyzed by electrophoresis on 0.8% agarose gels with 0.5X TBE buffer at 100 volts for approximately 1 hr, then stained with EtBr to visualize DNA bands under ultraviolet light.

## Competing interests

The author(s) declare that they have no competing interests.

## Authors' contributions

CPR participated in the design of the study, carried out the work with the insects, coordinated the project and assisted in the molecular analysis and drafting of the manuscript. JPB conceived the study, designed and supervised the experimental work and drafted the manuscript. All authors read and approved the final manuscript.

## References

[B1] Raina AK, Adams JR (1995). Gonad-specific virus of corn earworm. Nature.

[B2] Hamm JJ, Carpenter JE, Styer EL (1996). Oviposition day effect on incidence of agonadal progeny of *Helicoverpa zea *(Lepidotera: Noctuidae) infected with a virus. Ann Entomol Soc Am.

[B3] Burand JP, Miller LK, Ball LA Ball (1988). Nudiviruses. The Insect Viruses.

[B4] Burand JP, Lu H (1997). Replication of a gonad-specific virus in TN-368 cells in culture. J Invertebr Pathol.

[B5] Lu H, Burand JP (2001). Replication of the gonad-specific virus Hz-2V in Ld652Y cells mimics replication *in vivo*. J Invertebr Pathol.

[B6] Lupiani B, Raina AK, Huber C (1998). Deveopment and use of a PCR assay for detection of he reproductive virus in wild populations of *Helicoverpa zea *(Lepidopera: Noctuidae). J Invertebr Pathol.

[B7] Raina AK, Adams JR, Lupiani B, Lynn DE, Kim W, Burand JP, Dougherty EM (2000). Further characterization of the gonad-specific virus of corn earworm, *Helicoverpa zea*. J Invertebr Pathol.

[B8] Rallis CP, Burand JP (2002). Pathology and ultrastructure of the insect virus, Hz-2V, infecting agonadal female corn earworms, *Helicoverpa zea*. J Invertebr Pathol.

[B9] Rallis CP, Burand JP (2002). Pathology and ultrastructure of the insect virus, Hz-2V, infecting agonadal male corn earworms, *Helicoverpa zea*. J Invertebr Pathol.

[B10] King LA, Possee RD (1992). The Baculovirus Expression Vector System: A Laboratory Guide.

